# Preparation and Characterization of Electrochemically Deposited Cu_2_O/ZnO Heterojunctions on Porous Silicon

**DOI:** 10.1021/acsomega.3c01438

**Published:** 2023-05-31

**Authors:** Alper Çetinel, Gokhan Utlu

**Affiliations:** Department of Physics, Ege University, Bornova, 35030 Izmir, Turkey

## Abstract

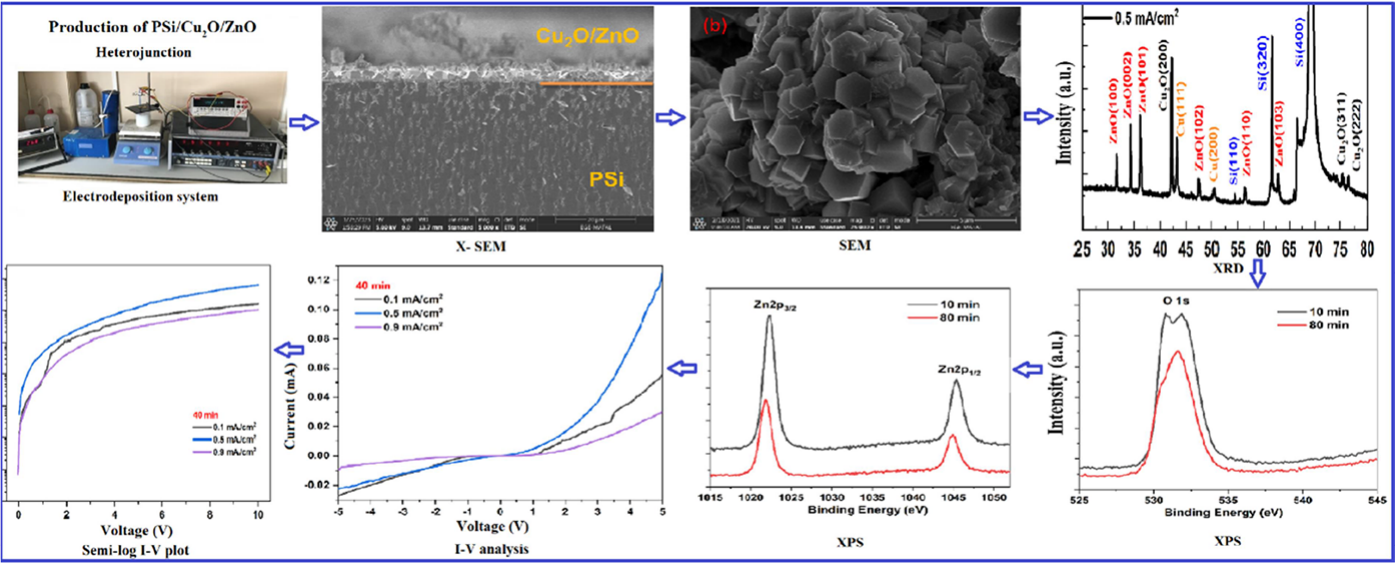

Cu_2_O/ZnO heterojunction was fabricated on porous silicon (PSi) by a two-step electrochemical deposition technique with changing current densities and deposition times, and then the PSi/Cu_2_O/ZnO nanostructure was systematically investigated. SEM investigation revealed that the morphologies of the ZnO nanostructures were significantly affected by the applied current density but not those of Cu_2_O nanostructures. It was observed that with the increase of current density from 0.1 to 0.9 mA/cm^2^, ZnO nanoparticles showed more intense deposition on the surface. In addition, when the deposition time increased from 10 to 80 min, at a constant current density, an intense ZnO accumulation occured on Cu_2_O structures. XRD analysis showed that both the polycrystallinity and the preferential orientation of ZnO nanostructures change with the deposition time. XRD analysis also revealed that Cu_2_O nanostructures are mostly in the polycrystalline structure. Several strong Cu_2_O peaks were observed for less deposition times, but those peaks diminish with increasing deposition time due to ZnO contents. According to XPS analysis, extending the deposition time from 10 to 80 min, the intensity of the Zn peaks increases, whereas the intensity of the Cu peaks decreases, which is verified by the XRD and SEM investigations. It was found from the *I*–*V* analysis that the PSi/Cu_2_O/ZnO samples exhibited rectifying junction and acted as a characteristical p-n heterojunction. Among the chosen experimental parameters, PSi/Cu_2_O/ZnO samples obtained at 0.5 mA current density and 80 min deposition times have the best junction quality and defect density.

## 1 Introduction

It has become increasingly common in optoelectronic device technology to combine the properties of different metal oxides (MOs) and/or alloys to produce and develop new materials with interesting and desirable properties.^1−3^ Among MOs, cuprous oxide-zinc oxide (Cu_2_O/ZnO) binary compound stands out with its remarkable electrical, optical, and thermal properties.^1,4^ In addition, they have several advantages such as low cost, material abundance, chemically stable, and low lattice mismatch (only 7.1%).^5^ Cu_2_O is a p-type metal-oxide semiconductor (MOS) with a band gap of 2 eV, a high absorption coefficient (10^2^–10^6^ cm^–1^), and a large exciton binding energy of 140 meV.^6^ ZnO is also a well-known n-type MOS, with a band gap of 3.3 eV, high electron mobility (120 cm^2^/V s), and binding energy of exciton (60 meV).^1^ Furthermore, the unique electrical and optical properties of these materials are important for solar cells and photodetector applications.^7,8^

Cu_2_O/ZnO can be fabricated using different deposition techniques such as magnetron sputtering,^9^ pulsed laser deposition,^10^ electrochemical deposition,^11^and so forth. Compared to other production methods, the electrochemical deposition method is a simple, fast, low-cost process. Moreover, it provides the ability to tailor the size, shape, and morphology of the nanostructures deposited under a set of well-controlled synthesis parameters. Controlling the size and shape of the nanostructures is technologically crucial due to the close correlation between these parameters and optical, electrical, and catalytic features.^12,13^ The most important problem in Cu_2_O/ZnO-based device applications is interface defects.^4,11^ In addition, obtaining high junction quality and low defect density is also a major challenge, so the experimental parameters must be well chosen. Fabricating good quality p-type ZnO semiconductor material is challenging due to oxygen vacancy and zinc interstitials in pure ZnO.^14^ In addition, the limited charge-transport properties of the ZnO/Cu_2_O heterojunction could be affected by the interface states, imposing a significant limit on the maximum thickness of the junction, resulting in poor power conversion efficiency, and thus, the formation of an effective p–n junction is the most important key factor.^4,15^

Up to now, although the growth and analysis of Cu_2_O/ZnO structures upon conventional substrates such as glass substrates, metallic substrates, and ITO- and FTO-substrates have been extensively studied,^16,17^ the physical, electrical, and optical features of the Cu_2_O/ZnO grown on porous silicon by electrodeposition have not been reported in the literature yet. However porous silicon (PSi) has been extensively studied for its unique photoluminescence properties. PSi’s importance has been increasing in the recent years because of its fascinating electrical properties.^13,18^However, due to the instability issue of porous silicon, developing stable porous silicon-based devices is a challenging process. For this reason, surface passivation with low-resistance, stable electrical contacts is required to increase the structural, optical, and electrical properties of the PSi structure and make it more stable as well as to develop porous silicon-based devices and their integration into electronic circuits.^13,19^ Accordingly, the purpose of our work is to study the properties of the PSi/Cu_2_O/ZnO structure by varying the electrochemical deposition parameters (time and current density) by providing better control over the electronic structure, crystallinity, and morphology of PSi/Cu_2_O/ZnO.

## 2 Materials and Methods

PSi layer was derived via anodization technique by using n-type silicon wafer with orientation of (100) and resistivity of 1–10 Ω cm as defined in recent works.^13,20,21^ Sigma Aldrich supplied all of the chemicals utilized in this study. Two-step deposition was performed to obtain PSi/Cu_2_O/ZnO nanostructures. First, PSi substrate (1 cm^2^ surface area) as an anode and a Pt wire as a cathode were used, respectively, in a solution containing 0.4 M CuSO_4_, 0.5 M boric acid (H_3_BO_3_), and 3 M lactic acid for the electrochemical deposition of Cu_2_O nanostructures. The pH of the solution was adjusted to 12 by adding 5 M KOH while maintaining a temperature of 65 °C. Cu_2_O structures were obtained for different deposition times (varied from 10 to 80 min) at current densities of 0.1, 0.5, and 0.9 mA/cm^2^. Then, the samples were cleaned with distilled water. In the second step of electrochemical deposition, ZnO deposition was performed under the same current densities and deposition times in an electrolyte composed of an aqueous solution of 0.08 M Zn(NO_3_)_2_·6H_2_O and 0.05 M C_6_H_12_N_4_. The electrolyte temperature and pH were 85 °C and 5.1, respectively. Finally, all samples were cleaned by distilled water.

Surface properties of PSi/Cu_2_O/ZnO were analyzed via field emission scanning electron microscopy (FE-SEM, QuantaFEG). Patterns of X-ray diffraction (XRD) of PSi/Cu_2_O/ZnO were obtained by a Panalytical Empyrean diffraction system employing CuKα radiation (λ = 0.15418 nm). Composition analysis was carried out by X-ray photoelectron spectrometry (XPS; Thermo Scientific K-Alpha) in the depth direction of the Cu_2_O/ZnO deposited on porous silicon. The *I*–*V* analysis was carried out via a Keysight B2901A source meter (SMU) between −5 and +5 V in a dark environment at ambient temperature (300 K).

## 3 Results and Discussion

**Figure 1:**
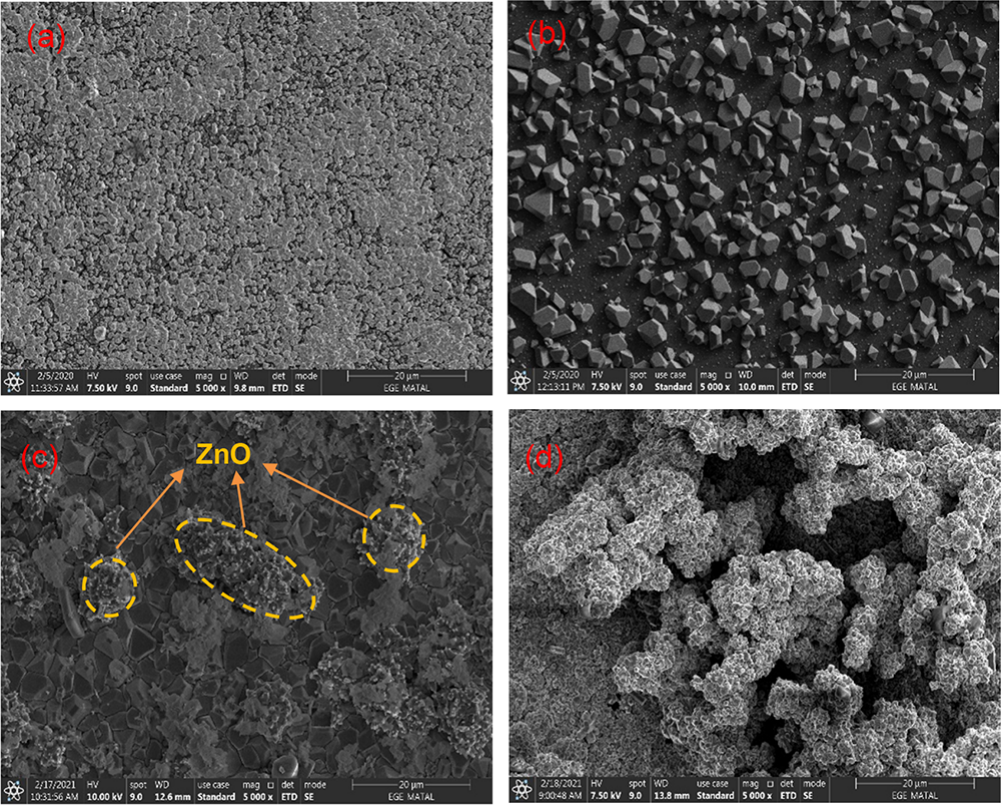
Surface FE-SEM analysis results of PSi/Cu_2_O/ZnO deposited at current density of 0.1 mA/cm^2^. (a) 10 min, (b) 20 min, (c) 40 min, and (d) 80 min.

**Figure 2:**
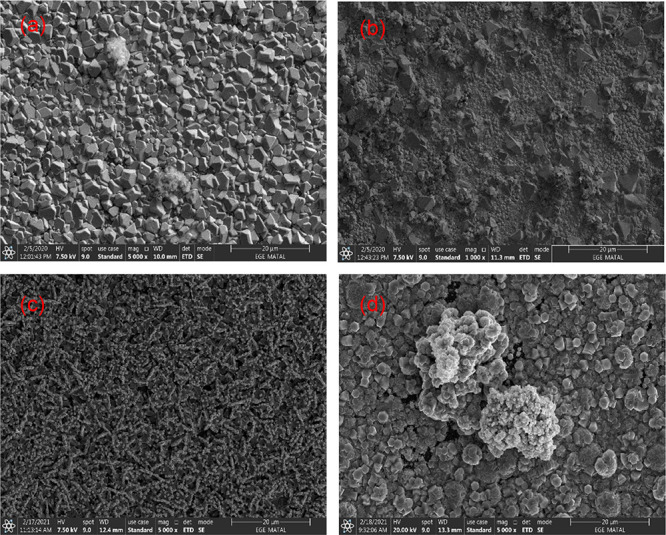
Surface FE-SEM analysis results of PSi/Cu_2_O/ZnO deposited at current density of 0.5 mA/cm^2^. (a) 10 min, (b) 20 min, (c) 40 min, and (d) 80 min.

**Figure 3:**
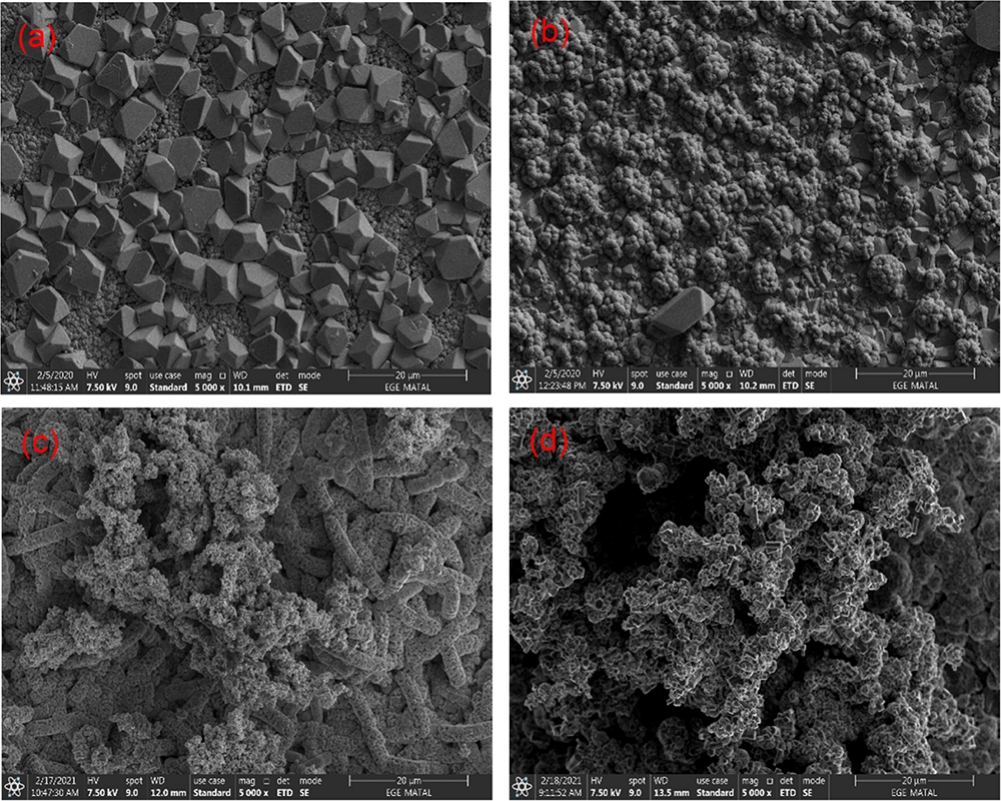
Surface FE-SEM analysis results of PSi/Cu_2_O/ZnO deposited at current density of 0.9 mA/cm^2^. (a) 10 min, (b) 20 min, (c) 40 min, and (d) 80 min.

**Figure 4:**
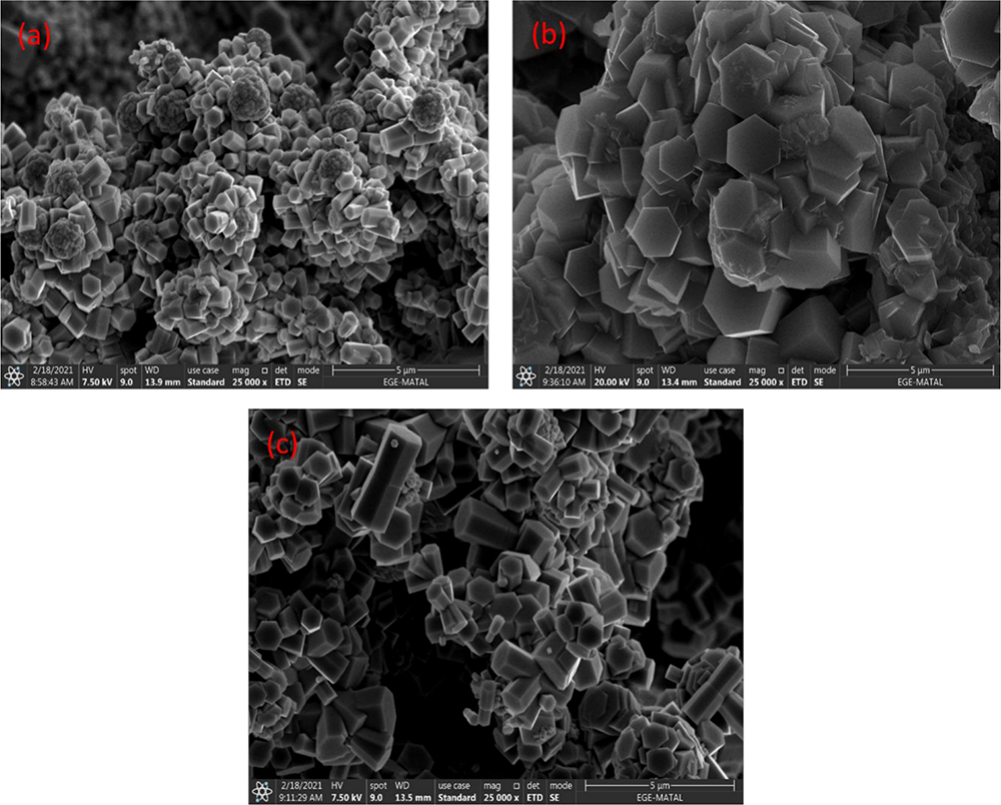
SEM images of PSi/Cu_2_O/ZnO prepared at 80 min deposition time for (a) 0.1 mA/cm^2^, (b) 0.5 mA/cm^2^, and (c) 0.9 mA/cm^2^.

Morphological and compositional properties of samples were analyzed by FE-SEM and EDX analyses. Figures 1^2^,3 indicate the surface properties of Cu_2_O/ZnO nanostructures prepared for different deposition times and current densities. The morphological properties of PSi/Cu_2_O/ZnO synthesized at a constant current density of 0.1 mA/cm^2^ are shown in Figure 1. As seen in Figure 1, for 10 min, Cu_2_O nanoparticles with the average diameter of ∼750 nm is obtained. At low current density and deposition times, especially in samples of 10–20 min, there are places where the substrate is exposed without a coating which is seen from the SEM analysis. This result shows that low current density and deposition times are not sufficient for Cu_2_O and ZnO deposition. On increasing the time to 20 and 40 min, Cu_2_O particles show truncated pyramidal and octahedral morphology (about 2.5–3 μm). SEM analysis revealed that no or small amount of ZnO nanoparticles was observed in the 10 and 20 min deposition times, whereas as seen in Figure 1c, spherical ZnO nanoparticles given in dashed circle formed around Cu_2_O nanostructures in the 40 min deposition time. For 80 min, it was observed that a film layer consisting of ZnO nanorods densely grew on the PSi/Cu_2_O surface. As seen from Figure 2, for 10 min, more ZnO nanostructures formed between polyhedral and pyramid Cu_2_O at 0.5 mA/cm^2^. When compared with 10 min deposition time at 0.1 mA/cm^2^, larger pyramid-like Cu_2_O structures with the average diameter around 2.5 μm were obtained for 10 min at 0.5 mA/cm^2^. When the time is increased to 20 min, Cu_2_O/ZnO nanostructures spread all over large areas on the surface and composed a film. As can be seen from Figure 2c, it was found that for 40 min deposition time, the ZnO nanorods clustered on the Cu_2_O nanoparticles and surrounded them in the shape of a belt. For 80 min, ZnO nanorods deposited more densely on the PSi/Cu_2_O surface at 0.5 mA/cm^2^. The largest octahedral and pyramid-like Cu_2_O structures were observed in Figure 3 when the current density was increased from 0.5 to 0.9 mA/cm^2^. For 10 min, the ZnO nanostructures are tiny and inhomogeneous around the octahedral and pyramidal shaped Cu_2_O structures (mean diameter 6.5 μm), though for 20 min deposition time, bigger ZnO nanoparticle clusters are detected on the surface (Figure 3b). SEM analysis indicate that with the increase in deposition time from 20 to 80 min, an intense ZnO deposition occured on Cu_2_O structures as observed at 0.1 and 0.5 mA/cm^2^. ZnO nanorod structures were observed in 80 min samples at all current densities (0.1, 0.5, and 0.9 mA/cm^2^). The SEM image obtained by zooming is also given in Figure 4. We can summarize the microstructure as follows:

When the current density and deposition time increase, the crystals look different just because of their different facet shapes, which varies with growth orientation. It can be seen from SEM analysis (Figures 1^2^,3) that there are pyramid-like Cu_2_O structures at low current density and low deposition times. As the current density and deposition time increase, deposition of ZnO occurs on these structures (especially short rod structures are formed depending on the growth direction).

**Figure 5:**
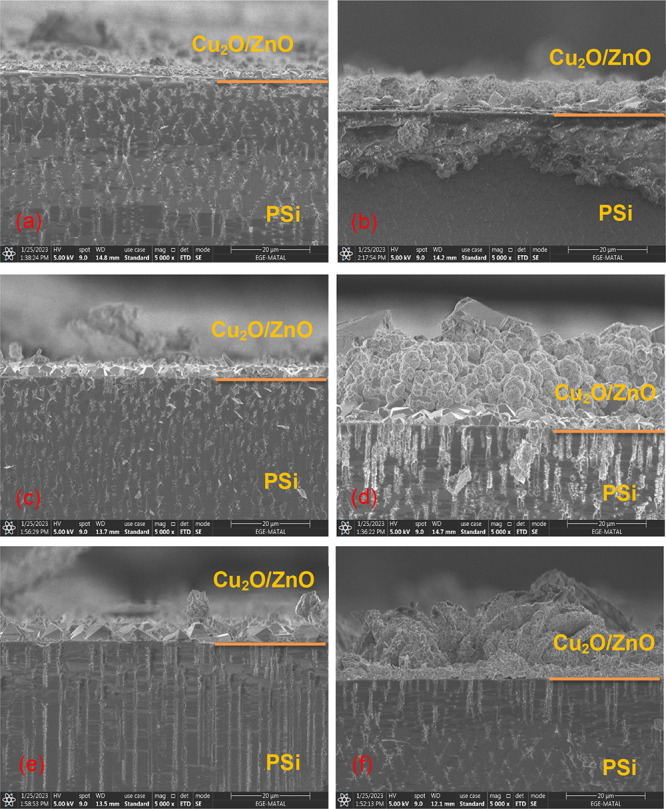
Cross-section FE-SEM images of PSi/Cu_2_O/ZnO prepared with different current densities and deposition times for 0.1 mA/cm^2^ (a) 10 min and (b) 80 min, for 0.5 mA/cm^2^ (c) 10 min and (d) 80 min, and for 0.9 mA/cm^2^ (e) 10 min and (f) 80 min.

From SEM analysis, it was understood that there was no significant effect of current density on the shapes of Cu_2_O nanostructures at constant deposition time, but the dimensions and shapes of ZnO nanostructures were significantly affected. It was observed that with the increase of current density from 0.1 to 0.9 mA/cm^2^, ZnO nanoparticles thickened and showed more intense deposition on the surface. The cross-sectional SEM (X-SEM) images of PSi/Cu_2_O/ZnO nanostructures prepared under different conditions are presented in Figure 5. The X-SEM images show that the film thickness formed on the surface increases with increasing deposition time. It has also been discovered that certain channels are continually loaded from the bottom of the pore to the PSi surface, while others are unfilled or partially loaded. The second occurrence is attributed to the bottleneck effect,^22,23^ which occurs when the pore mouth closes until it has been entirely loaded with the Cu_2_O nanoparticles. This could be caused by the closing of the pore mouth owing to size of the particle, which increases with the current density. It is worth noting that throughout the cleavage process, some cluster of nanoparticles can fall from the surface. X-SEM analysis further shows that the film thickness of the Cu_2_O/ZnO layer increases with increasing duration from 10 to 80 min. As seen in Figure 4a, with increasing deposition time, the film thicknesses of Cu_2_O/ZnO layer increase from 2 to 10 μm for 0.1 mA/cm^2^, from 3 to 18 μm for 0.5 mA/cm^2^, and from 5 to 15 μm for 0.9 mA/cm^2^.

**Figure 6:**
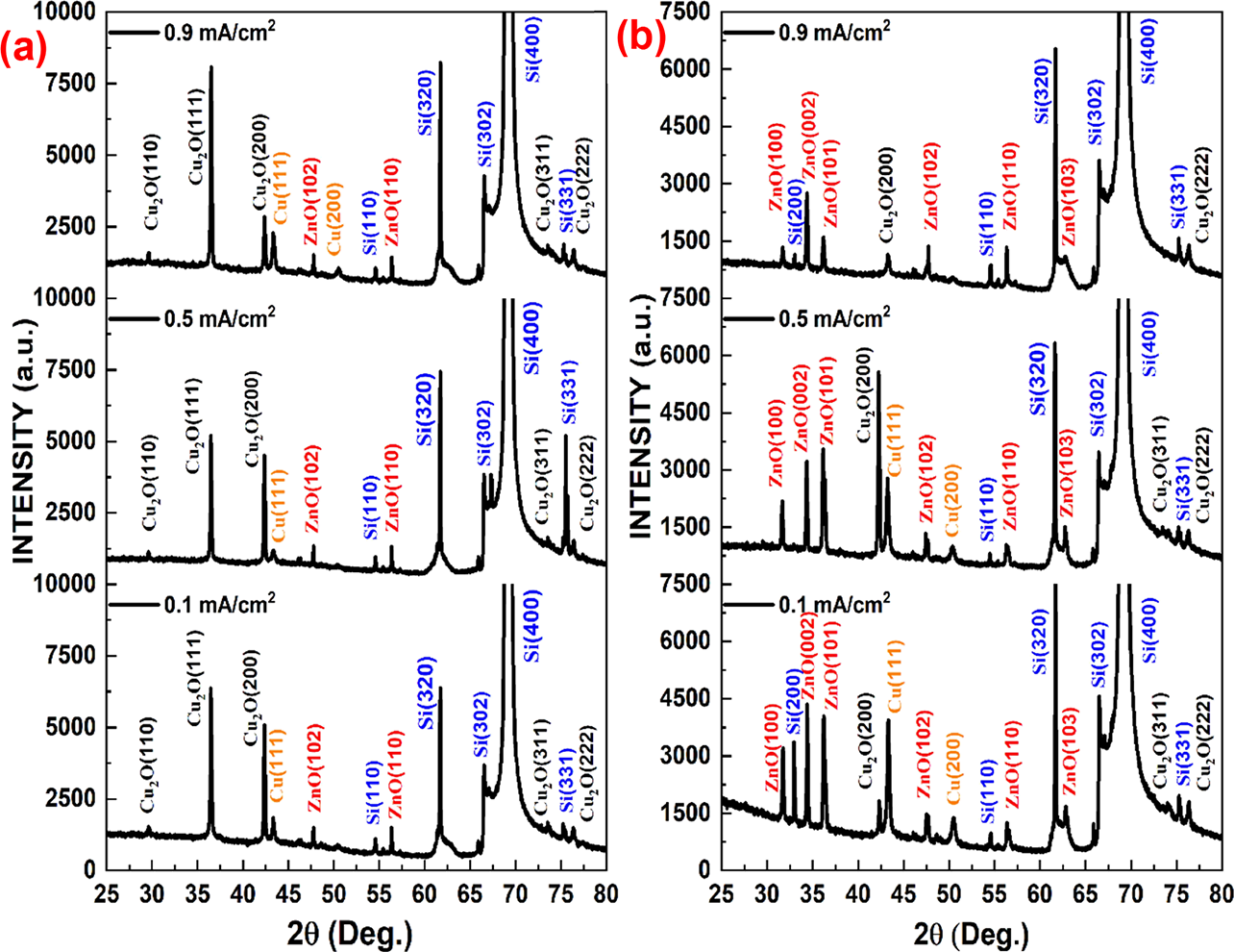
XRD patterns of PSi/Cu_2_O/ZnO nanostructures with different deposition times. (a) 10 and (b) 80 min.

The PSi/Cu_2_O/ZnO samples were also investigated via XRD to determine the purity of samples. The impact of deposition duration and current density on the XRD pattern of Psi/Cu_2_O/ZnO nanostructures is shown in Figure 6. In the case of 10 min deposition time, the diffractogram is dominated by Cu_2_O (Figure 6a). As seen in Figure 6, the peaks at 2θ of 29.59°, 36.45°, 42.34°, 61.66°, 73.55°, and 77.41° represent (110), (111), (200), (220), (311), and (222), respectively, corresponding the standard card of Cu_2_O (JCPDS card No. 01-078-2076).^13,24^ Besides the Cu_2_O XRD pattern, the peaks detected at 2θ values of 33.0°, 54.5°, 61.7°, 67.0°, 69.7°, and 75.4° match the (200), (110), (320), (302), (400), and (331) directions of the silicon substrate, respectively.^13,25,26^ Apart from Cu_2_O and Si, weak peaks at 2θ = 43.4° and 50.4° are known as (111) and (200) planes of metallic Cu.^13,27^ It should be noted that the amount of OH^–^ in the solution may affect the metallic copper formation in the structure.^13^ According to XRD analysis, Cu_2_O nanostructures are generally polycrystalline, cubic, and preferentially oriented along the plane of (111) for 10 min. Some high Cu_2_O peaks were found in the PSi/Cu_2_O/ZnO sample prepared for 10 min, but those intensities were reduced with increasing deposition time due to increasing ZnO content. XRD patterns of the PSi/Cu_2_O/ZnO sample prepared for 80 min reveal that the crystal structure of ZnO phases is polycrystalline hexagonal wurtzite with (100), (002), (101), (102), (110), and (103) orientations corresponding to 2θ = 31.76°, 34.41°, 36.23°, 47.50°, 56.56°, and 62.84° according to JCPDS card No. 01-074-0534.^17,21,28^ It has been also found that ZnO nanostructures have a polycrystalline, hexagonal wurtzite crystal structure, and its preferential orientation changed from (102) to (002) with increasing deposition time. This might be attributed to the fact that ZnO deposition on the Cu_2_O nanostructure increased with increasing deposition time.

Average crystal size was calculated from the dominant peaks (111) and (002) of PSi/Cu_2_O/ZnO samples by the Scherrer formula:^29^
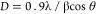
1where β, λ, and θ are the full width at half-maximum, the wavelength of X-ray, and the diffraction angle, respectively. The wavelength of the Cu K_α_ beam was taken as 1.54 Å. The calculated crystal sizes are listed in Table 1.

**Table 1:** Influence of Time and Current Density on the Crystallite Size of Cu_2_O/ZnO

current density (mA/cm^2^)	deposition time (min)	Cu_2_O	ZnO
		*D* (nm)	*D* (nm)
0.1	10	100.05	
	80	81.21	100.05
0.5	10	82.00	
	80	73.23	79.46
0.9	10	81.53	
	80	52.46	71.01

Table 1 indicates that when the current density increases, the average size of Cu_2_O crystallite declines. According to Çetinel^13^ and Ribic-Zelenovic et al.,^30^ the increase in lattice strain and nucleation sites causes the decrease in the size of the crystallite. They pointed out that the increase in the current/voltage reduces the crucial radius of the nucleus and the number of atoms that make up the nucleus, leading to faster nucleation and the creation of smaller crystals.^13,30^

**Figure 7:**
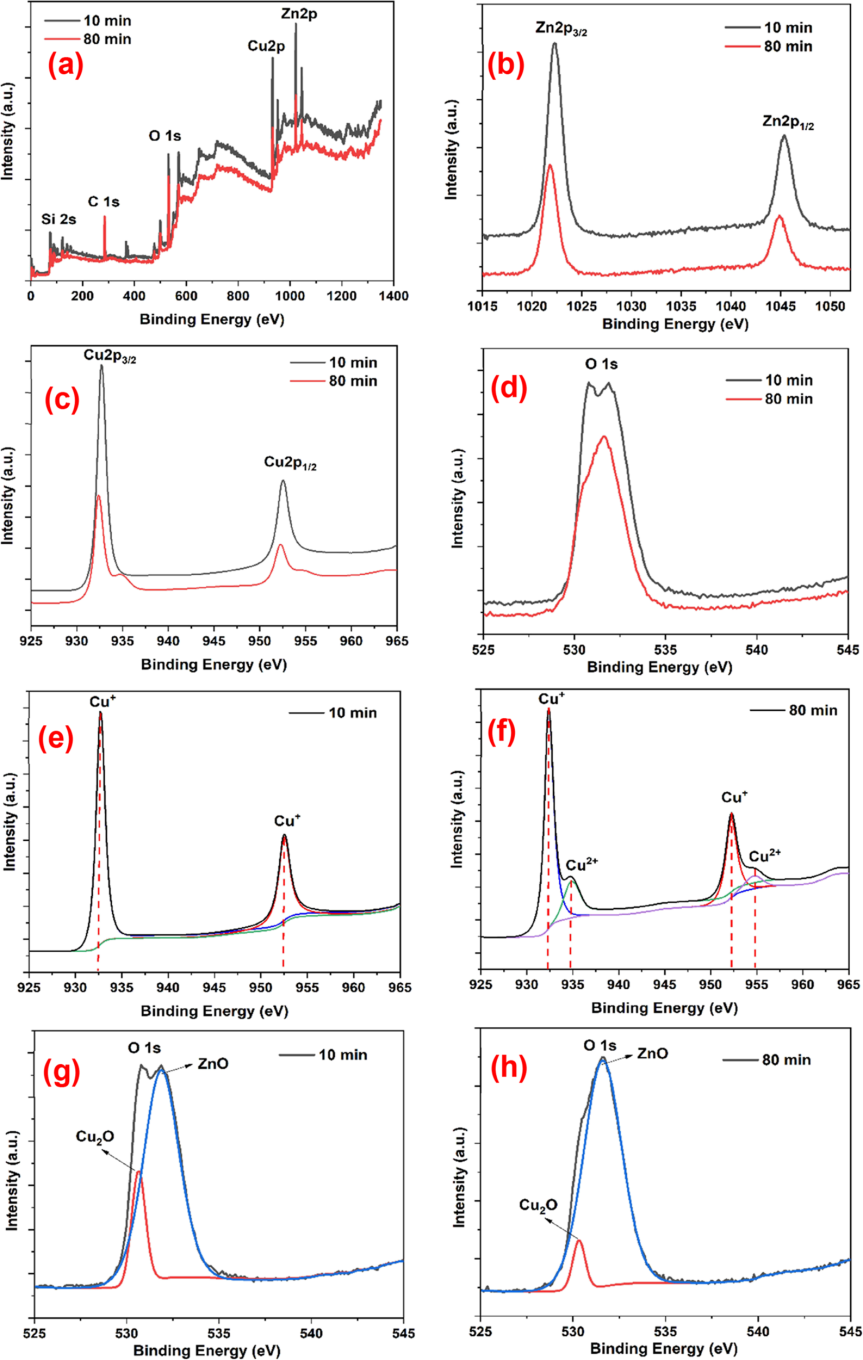
(a) XPS survey spectra of samples fabricated at 0.5 mA/cm^2^ for deposition times of 10 and 80 min. (b) Zn 2p region of XPS spectra, (c) Cu 2p region of XPS spectra, (d) O 1s region of XPS spectra, (e) XPS spectra of Cu 2p for the sample deposited for 10 min, (f) XPS spectra of Cu 2p for the sample deposited for 80 min, (g) XPS spectra of O 1s for the sample deposited for 10 min, and (h) XPS spectra of O 1s for the sample deposited for 80 min.

XPS analysis of PSi/Cu_2_O/ZnO samples was carried out to identify the surface atomic states. Figure 7 shows the XPS spectra obtained on PSi/Cu_2_O/ZnO samples prepared for 10 and 80 min deposition times. While Figure 7a represents the survey scan peaks for elemental Zn, Cu, and O, their high-resolution spectra are shown in Figure 7b–h. Because of spin–orbit coupling, the Zn and Cu peaks form as doublets of 2p_3/2_ and 2p_1/2_, respectively. For higher energy range, the peaks at 1045.3 and 1022.3 eV could be linked to Zn 2p_1/2_ and Zn 2p_3/2_, respectively, observed for Zn element (Figure 7b). The spin– orbit separation is about 23.0 eV. The peak of 2p_3/2_ detected at 1022.3 eV could be ascribed to the presence of Zn^2+^.^24,31,32^ Both Zn 2p spin–orbit components can be deconvoluted into a single peak located at 1022.3 and 1045.3 eV. In Figure 7c, two important peaks were detected at 952.6 eV for Cu 2p_1/2_ and 932.7 eV for Cu 2p_3/2_ (19.9 eV peak splitting) in the lower energy range, which belonged to Cu, which is consistent with the literature.^24,31−33^ While the well-known Cu^2+^ satellites around 942 and 962 eV, a clue to the existence of CuO, were not detected for 10 min deposition time (Figure 7e), for 80 min deposition time, there were weak satellite peaks at around 934 and 954 eV signifying the presence of the chemical state of Cu^2+^ (Figure 7f). As stated in the study by Han et al.,^34^ Cu^2+^ peaks in Cu_2_O films can be caused by two sources: oxidation of the outmost surface of Cu_2_O films kept in the ambient environment and Cu^2+^ adsorbed on unstable Cu vacancy sites during electrodeposition. According to XPS analysis, when the deposition time increases to 80 min, the Zn peak intensity increases, whereas the intensity of the Cu peaks decreases, which is supported by the XRD and SEM investigations. In addition, as can be seen in Figure 7d,g,h, the asymmetric O 1s peak observed in the PSi/Cu_2_O/ZnO samples decomposes into different coupling peaks. The highest energy peak at 531.88 eV originates from the oxygen atoms that are linked to Zn atoms (Zn–O) in the ZnO structure. Another peak at 530.60 eV comes from Cu–O bonds in the Cu_2_O structure. Moreover, there is a peak at 531.38 eV resulting from the oxygen (O–O) bonds present in PSi/Cu_2_O/ZnO.^7,9,35^

**Figure 8:**
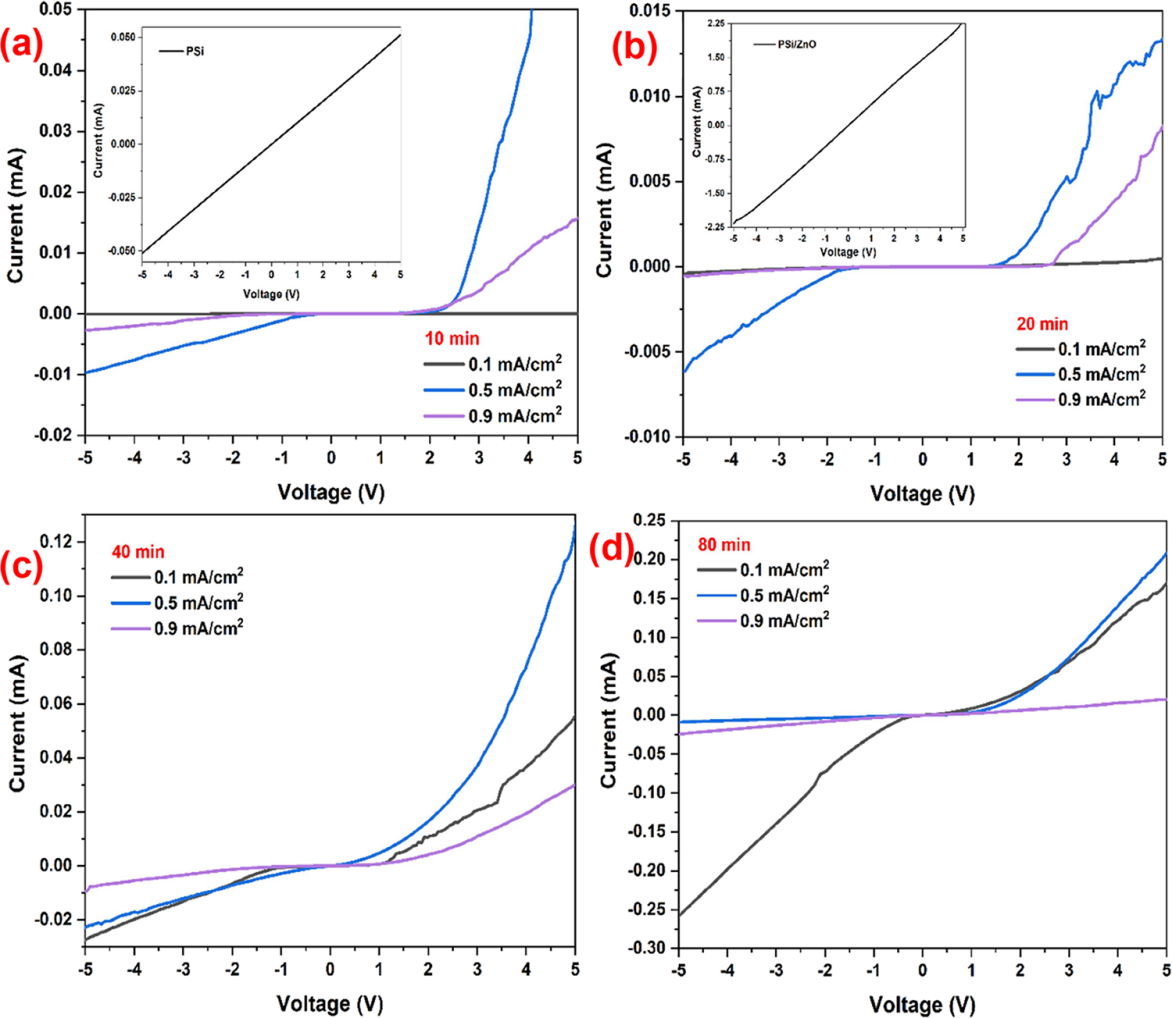
Current–voltage analysis of the PSi/Cu_2_O/ZnO samples in the dark environment at 300 K for the deposition time of (a) 10 min, (b) 20 min, (c) 40 min, and (d) 80 min.

**Figure 9:**
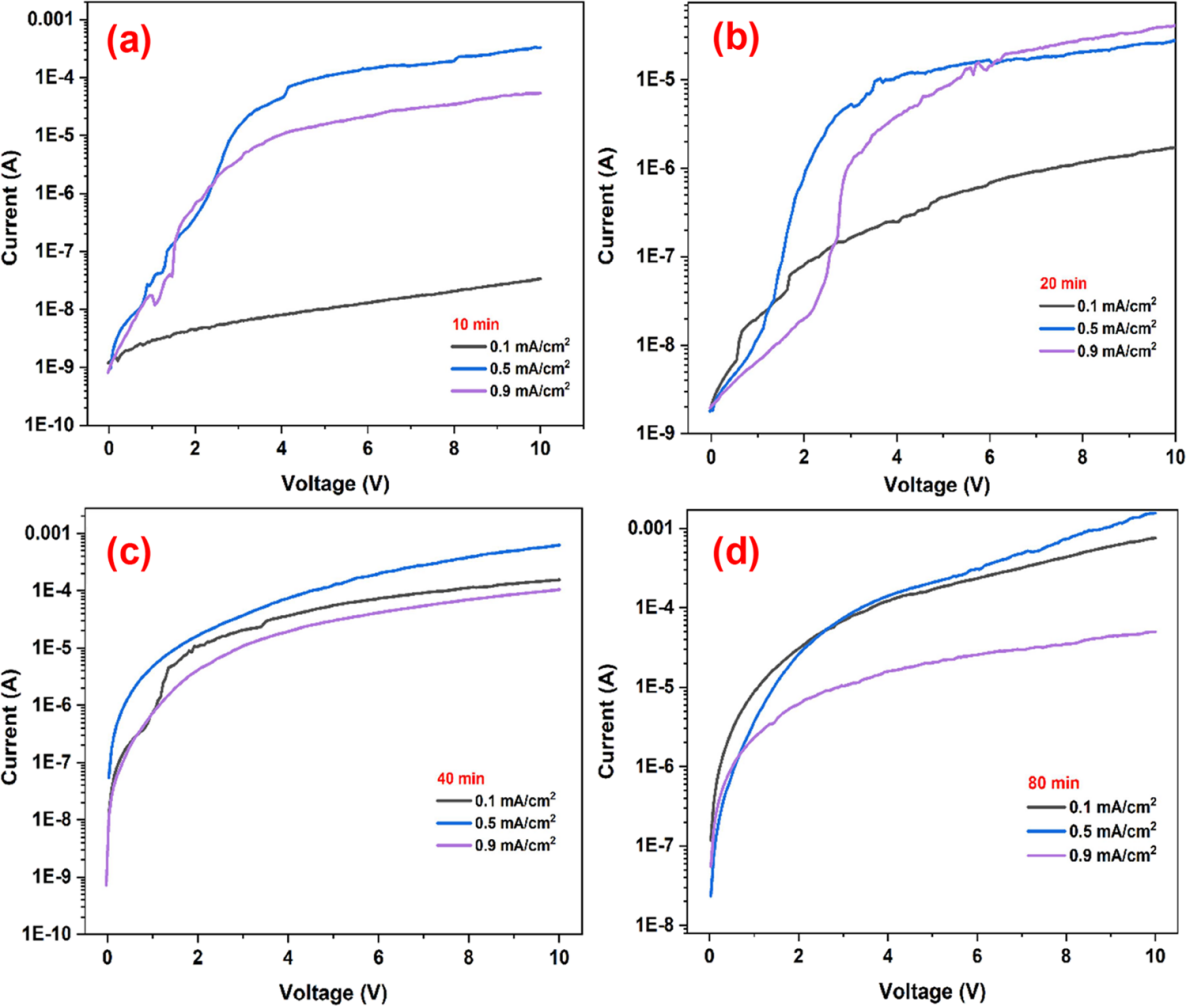
Semilog *I*–*V* plot of the PSi/Cu_2_O/ZnO heterojunction in the dark environment at 300 K for the deposition time of (a) 10 min, (b) 20 min, (c) 40 min, and (d) 80 min.

Electrical properties of PSi/Cu_2_O/ZnO samples were determined at 300 K under reverse and forward bias cases. Typical *I*–*V* for PSi/Cu_2_O/ZnO heterojunctions in both semilog and linear scales are represented in Figures 8 and 9. It is clearly seen from Figure 8 that PSi/Cu_2_O/ZnO heterojunctions reveal rectifier property and act as a typical p-n junction.^36−38^ The rectifying nature of the heterojunction was stable regardless of the deposition conditions. In reverse bias, the current decreases up to nA values due to the tunneling of electrons from the crystal silicon (c-Si) to the PSi/Cu_2_O structure. In forward bias, the achieved asymmetric characteristic in the *I*–*V* curve indicates the effective p-n heterojunctions between the p-Cu_2_O and n-ZnO layers. It is also worth noting that the PSi and PSi/ZnO samples exhibited a linear *I*–*V* characteristic ranging from −5 to +5 V (inset fin Figure 8), indicating that the p-n feature derives from the ZnO/Cu_2_O heterojunctions.

The semilog *I*–*V* plot was used to get the significant characteristics of the heterojunction diode, such as saturation current (*I*_0_), barrier height (φ_b_), and ideality factor (*n*), presented in Table 2 calculating from the well-known diode equation:^37^
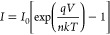
2
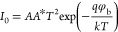
3where *I*_0_ is the saturation current, *k* is the Boltzmann constant, *q* is the electronic charge, *T* is the absolute temperature in K (at room temperature, 300 K), *V* is the bias voltage, *A* and A* are the effective diode area and Richardson constant (112 A/cm^2^ K^2^ for n-type Si), respectively.^18^Eq 3 may be used to calculate the φ_b_ using the *I*_0_ data. According to Hussain et al.,^39^ larger ideality factor values could be related to the oxide layer on the PSi/Cu_2_O surface, electrons and holes recombination in the depletion zone, interfacial states, or series resistance. According to SEM analysis, Cu_2_O nanostructures with a truncated pyramid shape with a random distribution have been generated on PSi in 10 min. For 80 min, as well as Cu_2_O deposition, ZnO nanoparticles aggregated and expanded both from the PSi surface and Cu_2_O nanostructures. For the current densities of 0.1 and 0.9 mA/cm^2^, the obtained Cu_2_O/ZnO layer on PSi is not ideal for the p-n junction. Higher ideality factor values for these samples might be attributable to a variety of factors such as inadequate film coverage, poor adherence to the substrate, and nonconducting grain boundaries.^12,13,40^ The low current value is also related to the high resistance at the PSi/Cu_2_O junction. At the current density of 0.5 mA/cm^2^, the ideality factor (*n*) value of PSi/Cu_2_O/ZnO samples decreased from 10 to 80 min. It has been also found that among the experimental parameters selected to produce the nanostructure, the sample fabricated at 0.5 mA/cm^2^ for 80 min has the lowest ideality factor (*n* = 1.36) due to a space charge effect at the grain boundary or interface.^41^ Thus, it is expected that the samples fabricated at the current density of 0.5 mA/cm^2^ will make a great contribution to advanced technological applications and literature as a functional material whose structural and electrical properties can be adjusted and which is easy to adapt to existing silicon technology.

**Table 2:** Electrical Properties of PSi/Cu_2_O/ZnO Heterojunction Derived from *I*–*V* Measurement [Ideality Factor (*n*), Saturation Current (*I*_0_), and Barrier Height (φ_b_)]

sample		*n*	*I*_0_ (A)	φ_b_ (eV)
0.1 mA/cm^2^	10 min	3.63	1.62 × 10^–9^	0.93
	20 min	3.90	1.47 × 10^–8^	0.92
	40 min	3.95	1.34 × 10^–6^	0.85
	80 min	3.80	6.94 × 10^–6^	0.75
0.5 mA/cm^2^	10 min	2.75	3.34 × 10^–8^	0.95
	20 min	1.98	4.57 × 10^–8^	0.93
	40 min	1.54	3.44 × 10^–6^	0.76
	80 min	1.36	3.23 × 10^–6^	0.80
0.9 mA/cm^2^	10 min	5.90	2.59 × 10^–8^	0.91
	20 min	5.30	6.33 × 10^–9^	0.93
	40 min	5.52	6.37 × 10^–7^	0.83
	80 min	5.14	2.00 × 10^–6^	0.78

## 4 Conclusions

In this study, an economical two-step electrochemical deposition approach was used to create Cu_2_O/ZnO nanoparticles with varied durations and current densities. SEM examination indicated that the Cu_2_O structures in the octahedron and truncated pyramid shapes were obtained, while ZnO nanostructures changed from spherical to hexagonal nanorods depending on the time and current density of the deposition method. XRD and XPS analyses showed that the structure had high crystallinity. As a result of the *I*–*V* analysis of PSi/Cu_2_O/ZnO heterojunctions in the dark environment at room temperature (300 K), it was found that the samples have rectifier features and act as a p-n junction. It has been also determined that among the experimental factors chosen for Cu_2_O/ZnO, the best parameters for the heterojunction diode and good rectifying properties were obtained at 0.5 mA/cm^2^.
